# An intimate partner violence prevention intervention for men, women, and couples in Ethiopia: Additional findings on substance use and depressive symptoms from a cluster-randomized controlled trial

**DOI:** 10.1371/journal.pmed.1003131

**Published:** 2020-08-18

**Authors:** Jessica Leight, Negussie Deyessa, Fabio Verani, Samuel Tewolde, Vandana Sharma

**Affiliations:** 1 International Food Policy Research Institute, Washington, DC, United States of America; 2 Ethiopian Public Health Association, Addis Ababa, Ethiopia; 3 Department of Preventive Medicine, School of Public Health, Addis Ababa University, Addis Ababa, Ethiopia; 4 CARE, New York, United States of America; 5 EngenderHealth, Addis Ababa, Ethiopia; 6 Harvard T.H. Chan School of Public Health, Boston, Massachusetts, United States of America; Harvard Medical School, UNITED STATES

## Abstract

**Background:**

Intimate partner violence (IPV) is linked to substance use by male perpetrators and is associated with an increased risk of depression for women who experience violence. Unite for a Better Life (UBL) is a gender-transformative intervention delivered to men, women, and couples in Ethiopia; previous evidence demonstrated the intervention significantly reduced experience of and perpetration of IPV when delivered to men and led to more equitable household task-sharing when delivered to men and couples. The aim of this analysis is to assess engagement in the UBL intervention and to examine the relationship between random assignment to the intervention and men’s past-year substance use and women’s reported depressive symptoms as measured at the individual level.

**Methods and findings:**

A sample of 64 villages in Gurague zone, Ethiopia, was randomly allocated to 4 arms (men’s UBL, women’s UBL, couples’ UBL, or control). In each village, 106 households were randomly sampled, and households in the intervention arms were invited to participate in UBL, consisting of 14 sessions delivered by trained facilitators. Households in the control arm were offered a short educational session on IPV. Descriptive data on participant engagement in the intervention are reported, and outcomes assessed in an intention-to-treat (ITT) analysis include male use of substances (alcohol and khat) and women’s depressive symptoms as measured by the Patient Health Questionnaire (PHQ-9). Results from both adjusted and unadjusted specifications are reported, the latter adjusting for baseline covariates including age, education level, marriage length, polygamy, socioeconomic status, months between intervention and endline, and the baseline level of the outcome variable. The baseline sample includes 6,770 respondents surveyed in 2014–2015, and follow-up data were available from 88% of baseline respondents surveyed in 2017–2018; the majority of respondents report no education, and 61% are Muslim. Respondents reported high attendance rates and engagement in the intervention. In addition, there was evidence of a significant reduction in frequent past-year alcohol intoxication self-reported by men (adjusted odds ratio [AOR] = 0.56, 95% CI 0.36–0.85, *p* = 0.007), and a significant increase in the probability of frequent khat use self-reported by men (AOR = 3.09, 95% CI 1.37–6.96, *p* = 0.007), both observed in the couples’ UBL arm at 24 months’ follow-up relative to the control arm. There was a significant increase in symptoms of moderate depression among women in the women’s UBL arm only (AOR = 1.65, 95% CI 1.13–2.41, *p* = 0.010), again relative to the control arm. There was no evidence of shifts in symptoms of mild or severe depression. The primary limitation of this study is the reliance on self-reported data around sensitive behaviors.

**Conclusions:**

The findings suggest that the UBL intervention was associated with a reduction in men’s use of alcohol when delivered to couples, but there was no evidence of a decrease in reported symptoms of depression among women in any experimental arm, and some evidence of an increase in symptoms of moderate depression in the women’s UBL arm. Further research should explore how to optimize IPV prevention interventions to target related risks of mental health and substance use.

**Trial registration:**

Clinicaltrials.gov NCT02311699; Socialscienceregistry.org AEARCTR-0000211.

## Abstract 2

In this analysis of additional findings from a cluster-randomized controlled trial, Jessica Leight and colleagues investigate the associations between an intervention to reduce intimate partner violence, and substance abuse and depressive symptoms in men and women in Ethiopia.

## Author summary

**Why was this study done?:**

- IPV is a major challenge across the globe, and it is closely linked to other public health challenges, including substance use and mental health.
- However, preventive interventions that seek to reduce IPV rarely analyze or report the effects of these programs on these other outcomes, particularly in developing countries.

**What did the researchers do and find?:**

- The authors conducted a cluster-randomized controlled trial in a sample of 64 villages in southern Ethiopia. The baseline sample included 6,770 respondents, and follow-up data were available from 88% of baseline respondents.
- Villages were randomly assigned to receive a gender-transformative intervention, UBL, targeting the reduction of IPV and associated risk behaviors; this intervention was delivered to women, men, or couples in 3 different experimental arms. A fourth experimental arm served as the control group.
- Substance use was assessed using reports by both men and women of male substance use, and depressive symptoms were assessed using the PHQ-9 administered to women.
- At endline, there was some evidence of a decrease in reported alcohol intoxication and an increase in reported khat use in the couples’ UBL arm, as reported by men only.
- There was no evidence of a decrease in reported symptoms of depression among women in any experimental arm and some evidence of an increase in symptoms of moderate depression in the women’s UBL arm.

**What do these findings mean?:**

- Interventions that are effective in reducing IPV may not necessarily be associated with changes in substance use or depression, and programs should consider including additional content targeting these challenges.
- Future research and programs should particularly explore the relationship between khat and IPV in contexts in which khat use is common and culturally significant.

## Introduction

Intimate partner violence (IPV) is a major challenge across the globe, and it has serious implications for the long-term health and well-being of women and their families [1]. In rural Ethiopia, the site of this study, existing evidence suggests that 49% of ever-partnered women have experienced physical violence by a partner at some point in their lives, and 60% have experienced sexual violence [2].

In addition, IPV is closely linked to other public health challenges, including substance use and mental health. A large body of literature argues that heavy alcohol consumption by men increases the risk of perpetrating IPV [3–6]. Women who experience IPV are themselves at increased risk of use of alcohol or other substances, linked to post-traumatic stress disorder engendered by violence [2,7].

In the East African context, the relationship between the use of khat and IPV is increasingly a focus of research and policy debates, though the evidence base is limited. Khat is a naturally occurring plant-based stimulant (scientific name *Catha edulis*) traditionally consumed via chewing [8]. In Ethiopia, khat cultivation and use has spread from its traditional base among Muslim communities, particularly in the Harar region, and is now widespread [9]. Evidence from East Africa suggests that use of khat as well as alcohol by male partners is associated with an increased risk of IPV in cross-sectional data [10,11], and consumption of both substances by men is identified by women as a risk factor for IPV [12]. Use of khat by women in Ethiopia is associated with a higher probability of experience of gender-based violence [13] and forced sex, including both partner and nonpartner rape [14]. Use of khat among men in Ethiopia and Somalia is also associated with an increased probability of violent behavior [15,16].

Preventive interventions that target consumption of alcohol and other substances have been identified as an important strategy to reduce IPV risk and associated health consequences [4]. However, there is little literature around the effect of interventions addressing the interrelated risks of substance use and IPV in developing country contexts. Previous evidence suggests that the Stepping Stones program, targeting enhanced sexual health for young men and women in South Africa, led to a significant reduction in alcohol use among men but not women [17], a finding that was replicated in a more recent evaluation of Stepping Stones in conjunction with a livelihood-strengthening intervention [18]. The Raising HIV Awareness in Non-HIV-Infected Indian Wives (RHANI) Wives intervention targeted women in urban Mumbai experiencing IPV and heavy spousal alcohol use, and the intervention included a focus on alcohol as a point of spousal conflict; however, changes in substance use patterns were not measured or reported [19]. A recent evaluation reported that an alcohol harm reduction intervention reduced exposure to interpersonal violence and engagement in sex work among a population of sex workers in Mombasa, Kenya [20]. Other evaluations of IPV-focused interventions have not analyzed substance use.

At the same time, serious mental health consequences of IPV have been widely identified in the literature, particularly depression and post-traumatic stress disorder; this may contribute to the identified global sex disparity in depression between women and men [1,21,22]. Cross-country evidence suggests that women who report at least 1 episode of IPV report significantly more emotional distress and suicidal thoughts and attempts relative to women with no history of abuse [23]. A recent systematic review and meta-analysis of cohort studies found 8 studies that showed a positive association between recent IPV and subsequent depression [24], and other systematic reviews have similarly reported evidence of bidirectional relationships between depression and IPV [25]. Additional research analyzing the relationship between mental health and IPV suggests that social support and assistance (both actual and perceived) that women receive from others within their social networks is a mediating factor between abuse and distress, leading to reduced negative psychological effects [26,27].

Despite this literature, evaluations analyzing IPV prevention programs—programs that frequently include group-based sessions that strengthen social interactions—often do not collect adequate data to assess effects on women’s well-being or depression. For example, a recent systematic review of structural interventions targeting IPV in low- and middle-income countries did not present any evidence on the effects of these interventions on depression or mental health [28]. Among the major randomized trials evaluating IPV prevention interventions, only 3 reported effects on depression or depressive symptoms: the Stepping Stones evaluation reported no effect on self-reported depression among men or women [17], Stepping Stones in conjunction with Creating Futures reported a decrease in self-reported depression among men [18], and a recent evaluation of a rapid response system seeking to prevent IPV in Ghana reported a decrease in reported depression among women [29].

This paper reports additional results from a randomized controlled trial (RCT) conducted in southern Ethiopia, analyzing the relationship between a gender-transformative, participatory intervention and male substance use and female mental health as measured approximately 24 months following the intervention. The Unite for a Better Life (UBL) program has the objective of reducing physical and sexual IPV and HIV risk behaviors and promoting healthier, more equitable relationships. The program comprises 14 in-person sessions delivered to groups of men, women, and couples in the context of the coffee ceremony, a traditional forum for community-based discussion in Ethiopia. Recently reported results suggest that the UBL intervention when delivered to men generated a large and statistically significant decline in women’s experience of past-year physical and/or sexual IPV as well as male perpetration of past-year sexual IPV, and physical and/or sexual IPV at approximately 24 months follow-up [30]. Though the intervention did not reduce IPV when delivered to women or couples, individuals in the men’s and couples’ arms show evidence of significant shifts toward gender equity in household decision-making and task-sharing, and individuals in all 3 arms exhibited significant change for a number of indices linked to HIV knowledge and risk-taking behaviors [30].

Given the evidence of the UBL intervention’s significant effects on IPV-related outcomes as well as its supportive group-based approach, it is plausible to hypothesize that the intervention may have also been associated with reductions in male use of substances, as well as enhancements in women’s mental health [1,3,4]. The objective of this paper is to assess social interaction and engagement with the UBL intervention and to examine the relationship between random assignment to the UBL intervention and past-year substance use by men and women’s reported depressive symptoms as measured at the individual level.

## Methods

### Study design

This paper reports on substance use and mental health outcomes measured in a 4-arm cluster-randomized controlled trial conducted in the Gurague zone of the Southern Nations, Nationalities and People’s Region (SNNPR) of Ethiopia between 2014 and 2018. The trial was a collaboration between the Abdul Latif Jameel Poverty Action Lab (J-PAL) at the Massachusetts Institute of Technology (MIT), the Addis Ababa University School of Public Health (AAU), the Ethiopian Public Health Association (EPHA), and EngenderHealth. The outcomes of interest presented in this paper were not prespecified as secondary outcomes in the trial registry; however, they were included in the original trial protocol (S1 Text, p. 9), and specified as additional outcomes to be analyzed in a pre-analysis plan registered prior to analysis (S2 Text, p.5).

In addition to a high prevalence of IPV as reported in the 2005 WHO Multi-country Study on Women’s Health and Domestic Violence [1], Ethiopia is characterized by high rates of substance use among men and depression among women. According to the 2016 Demographic and Health Survey (DHS), 46% of Ethiopian men aged 15–49 consumed alcohol at least once in the last year, and among this sample, 58% consumed alcohol at least once a week [31]. Khat use is also common; 27% of men reported using khat, and among this sample, 58% used it more than 6 days in the last 30 days [31]. There is also evidence of correlations between IPV, substance use, and mental health challenges: 81% of women reporting frequent alcohol intoxication by their spouses also reported experiencing IPV [31]. Evidence from a different cross-sectional survey conducted in our study region suggests a 12-month prevalence of depressive episodes of 4.8% among women and similarly reports a strong association between IPV and female depression [32].

This trial employed a cluster design including 64 villages (kebeles) in 4 districts (Mareko, Meskan, Silte and Sodo). The intervention of interest, described in more detail below, is a group-based gender-transformative intervention; accordingly, a cluster-level randomization is appropriate. Each kebele constituted a cluster, and the sampled kebeles were randomly selected from the full set of kebeles in these districts. Clusters were then randomly assigned to 1 of 4 study arms (UBL for women, UBL for men, UBL for couples, and control) utilizing a parallel randomization design, employing an equal allocation ratio and stratification at the district level. Randomization was conducted by the researcher team using a reproducible seed in Stata version 12.0. In addition, a further within-kebele randomization assigned each household with equal probability to the men’s survey subarm (in which the male spouse was surveyed at baseline) or the women’s survey subarm (in which the female spouse was surveyed at baseline).

All households within each cluster including a married or cohabiting couple in which the woman was between the ages of 18 and 49 years were eligible for inclusion in the trial. Within each cluster, 106 households were randomly selected using the roster of households maintained by community health workers and replaced if the household was found to be ineligible. In polygamous households, a wife was selected via simple random sampling for inclusion in the evaluation, and her age was used to determine eligibility. The identity of the individual surveyed at baseline was then determined by the village-level assignment to the men’s or women’s survey subarm.

In addition, 80% of individuals enrolled in the trial were randomly sampled for inclusion in the intervention in treatment villages, using an individual-level, within-village randomization protocol implemented by the research team. Those individuals in intervention communities not sampled for inclusion in the intervention were surveyed at baseline and endline in order to measure spillover effects of the intervention in a future analysis; this subsample is not included in this analysis. In the control communities, by contrast, the full sample of individuals surveyed at endline is included in this analysis.

This study is reported as per the Consolidated Standards of Reporting Trials (CONSORT) guideline (S1 CONSORT Checklist).

### Data collection

Data were collected from sampled participants at baseline from December 2014 to March 2015. Follow-up surveys were conducted with baseline respondents and their spouses between March 2017 and October 2017, approximately 24 months post-intervention. The addition of a short spousal survey was a modification to the protocol post-baseline, but follow-up data from spouses are not analyzed in this paper as data on substance use and depressive symptoms were not collected from spouses. To minimize attrition, additional endline data collection was conducted between January 2018 and March 2018. The trial concluded as planned 24 months post-intervention.

For both waves of data collection, a team of male and female Amharic-speaking enumerators recruited in the study districts administered paper-based surveys following 4 weeks of training on survey administration, ethics, interviewing skills, safety, and referral protocols. The questionnaires included modules on sociodemographic information, gender norms, household decision-making, substance use, HIV, IPV and mental health for women. A list of local medical, legal, and other relevant support services was given to respondents, and referrals for psychological support were provided.

Protocols around confidentiality as recommended in the WHO ethical and safety guidelines for IPV research were strictly followed [33]. In addition, respondents were referred to psychological support if they disclosed distress or mental health symptoms, and a directory of local support services was provided to all female respondents participating in the data collection.

Blinding of sampled individuals was not possible, as they were informed of their treatment assignment when invited to participate in the intervention (or, in the case of individuals in the control arm, a brief informational session). Data collection staff were blind to treatment assignment at baseline but at endline may have observed materials linked to the intervention assignments.

The study protocol was approved by the Committee on the Use of Humans as Experimental Subjects (COUHES) at MIT (protocol number 1211005333) and by the Institutional Review Board at the Addis Ababa University College of Health Sciences (protocol number 044/12/SPH). The trial was prospectively registered on clinicaltrials.gov (NCT02311699), and at the AEA registry (AEARCTR-0000211). Oral informed consent was obtained from all individuals enrolled in the trial prior to randomization.

### Intervention

This evaluation analyzes the impact of UBL, a gender-transformative intervention delivered via the Ethiopian coffee ceremony. EngenderHealth led the design of the curricula for the women’s, men’s and couple’s arms, and the delivery of the intervention was managed by AAU and EPHA. The program entailed 14 sessions delivered twice a week to groups including approximately 20 individuals. Accordingly, each cluster had 4 separate UBL groups running in parallel in the men’s and women’s arms and 8 groups in the couples’ arm. Each session was designed to be of 120–180 minutes’ duration, and included a coffee ceremony, discussion, and interactive activities focused on gender norms, sexuality, communication and conflict resolution, HIV/AIDS, and IPV. Facilitators encouraged active participation by all attendees and had opportunities to seek one-on-one feedback from attendees following each session.

Although the intervention curricula do not include specific sessions focused on substance use, the relationship between IPV and substance use is highlighted throughout the 14 sessions. In particular, the curricula emphasize the pressure men may experience to manifest masculinity via alcohol or khat use, strategies to resolve conflicts that arise due to excessive alcohol use by male partners, and the belief that alcohol or khat use causes or justifies violence. Identifying potential services for alcohol or khat dependency is included as a potential response to IPV. In addition, the logic model for the men’s intervention identified as an intermediate goal an increase in “knowledge about how the use of alcohol can affect [the] ability to express emotions in healthy ways.” The curricula placed a greater emphasis on the relationships between alcohol and violence and the adverse consequences of alcohol use, relative to use of khat and other drugs. This reflected a stronger ex-ante evidence base around the relationship between alcohol use and IPV in the literature; the evidence around relationships between khat and IPV is much more limited, and much of the literature is recent [10–16].

In the area of mental health, decreasing the prevalence of depression and stress was identified in the intervention logic models as a secondary health goal for all 3 intervention arms. In addition, given the evidence previously reported of the strong correlation between experience of IPV and mental health [21–25], an intervention that effectively targets IPV prevention may also be expected to have additional effects on women’s mental health.

Facilitators were recruited locally and extensively trained by the research team. In the women’s and men’s UBL arms, one same-gender facilitator led the intervention sessions, and in the couples’ UBL arm, groups were jointly moderated by one male and one female facilitator. Specific learning objectives were mapped out for each session and included describing and understanding gender norms and sexuality; identifying healthy and unhealthy relationship behaviors and understanding power within a relationship; strengthening intra-couple communication; understanding HIV and HIV prevention strategies; identifying violence and the consequences of violence; understanding concepts of sexual consent; and challenging violence. In communities in the control arm, an hour-long short informational session on IPV and HIV/AIDS prevention that was not designed to be participatory was offered.

### Outcomes

This analysis focuses on a number of outcomes. First, we report descriptive statistics on engagement in the UBL intervention (as reported by both the female and male respondents) in order to assess the level of social interaction associated with participation in the program, as well as continued social interaction with group members following the program’s completion. Second, we analyze additional outcomes of interest, including men’s substance use (as reported by both the female and male respondents) and women’s mental health (as reported by the female respondents). All outcomes are measured at the individual level approximately 24 months following intervention exposure.

Questions on intervention engagement were included in the surveys administered to both baseline respondents and their spouses at endline but are reported for the individuals invited to participate in the intervention in that arm: e.g., men in the men’s UBL arm, women in the women’s UBL arm, and both men and women in the couples’ UBL arm. This sample thus includes 2,470 women and 2,515 men. Detailed intervention questions were posed only to respondents who reported attending at least one intervention session. The variables are summarized in Table 1. The first variable of interest is the number of sessions reported attended. The second variable is a binary variable equal to one if the respondent reported s/he remembered some or all of what was discussed and zero otherwise. We also analyze binary variables equal to one if the respondent reported s/he shared information with other individuals and more than 5 individuals, respectively, and zero otherwise.

**Table 1:** Outcomes of interest.

Variable	Respondent	Definition
**Substance use**		
Frequent past-year alcohol use (men)	Women and men	Binary variable: Equal to one if respondent reports that over the previous 12 months, husband (for women) or self (for men) drank alcohol every day or nearly every day, or once or twice a week.
Frequent past-year alcohol intoxication (men)	Women and men	Binary variable: For respondents who report that husband (for women) or self (for men) ever drank over the last 12 months, equal to one if respondent reports that over the previous 12 months, husband (for women) or self (for men) was drunk most days or weekly.
Reports problems linked to own/husband’s alcohol use	Women and men	Binary variable: For respondents who report that husband (for women) or self (for men) ever drank over the last 12 months, equal to one if respondent reports problems linked to alcohol use (money problems, health problems, conflict with family or friends, problems with authorities, or other).
Frequent past-year khat use (men)	Women and men	Binary variable: Equal to one if respondent reports that over the last 12 months, husband (for women) or self (for men) used khat every day or nearly every day, or once or twice a week.
Reports problems linked to own/husband's khat use	Women and men	Binary variable: For respondents who report that husband (for women) or self (for men) ever used khat over the last 12 months, equal to one if respondent reports problems linked to alcohol use (money problems, health problems, conflict with family or friends, problems with authorities, or other).
**Mental health**		
Any depressive symptoms	Women	Binary variable: Equal to one if PHQ-9 score is greater than zero.
Symptoms of mild depression	Women	Binary variable: Equal to one if PHQ-9 score is greater than or equal to 5 and less than 10.
Symptoms of moderate depression	Women	Binary variable: Equal to one if PHQ-9 score is greater than or equal to 10 and less than 20.
Symptoms of severe depression	Women	Binary variable: Equal to one if PHQ-9 score is greater than or equal to 20.
Depressive symptoms affecting daily functioning	Women	Binary variable: Equal to one if the respondent reports that depressive symptoms interfere with daily functioning (at work, at home, or in interpersonal relations). This question is posed only to respondents who report at least one depressive symptom.

PHQ-9, Patient Health Questionnaire-9.

Continued program engagement is captured by a binary variable equal to one if the respondent reported s/he continues to meet with other UBL group members to discuss topics related to the program and zero otherwise. We also analyze a binary variable equal to one if the respondent reported s/he is highly satisfied with the UBL intervention (reported satisfaction at a level of 4 or 5 on a 5-point scale) and zero otherwise and a binary variable equal to one if s/he reported s/he is very likely to recommend the program to a friend (reported likelihood at a level of 4 or 5 on a 5-point scale, and zero otherwise).

The substance use module was administered to the original (baseline) female and male respondents at endline, a sample including 2,657 and 2,591 men. Both male and female respondents were posed questions about the frequency of alcohol consumption in the last 12 months by spouse/self; for individuals who reported any alcohol use, an additional question was posed about the frequency of drunkenness (described as such, using the appropriate Amharic translation) by spouse/self. Respondents also reported the frequency of khat use by spouse/self in the past year. For individuals who reported any alcohol or khat use by spouse/self, they were asked to report whether this use pattern generated any adverse consequences (challenges linked to money, health, conflict with family or friends, problems with the authorities, or any other problems).

The following variables are constructed, again as summarized in Table 1. Frequent past-year alcohol use is a binary variable equal to one if alcohol use by the male spouse over the past 12 months was reported every day or nearly every day or once or twice a week, and zero otherwise. Frequent past-year intoxication is a binary variable equal to one if intoxication by the male spouse was reported most days or weekly and zero otherwise, restricted to the sample reporting any past-year alcohol use. Frequent past-year khat use is a binary variable equal to one if khat use by the male spouse was reported every day or nearly every day or once or twice a week and zero otherwise. Two binary variables are constructed equal to one if problems reported to alcohol use or problems reported to khat use were reported and zero otherwise. Data on substance use as reported by both female and male respondents are analyzed separately.

The mental health module was administered only to the original (baseline) female respondents at endline, a sample including 2,657 women. For mental health outcomes, female respondents were administered the Patient Health Questionnaire-9 (PHQ-9) depression screening tool, a tool that has been previously validated in the Ethiopian context [34]. Consistent with existing guidelines in the literature, 3 binary variables are constructed from the PHQ-9 scores: a binary variable for reporting symptoms of mild depression (PHQ-9 score greater than or equal to 5 and less than 10), a binary variable for reporting symptoms of moderate depression (PHQ-9 score greater than or equal to 10 and less than 20), and a binary variable for reporting symptoms of severe depression (PHQ-9 score greater than or equal to 20) [35]. A binary variable is also constructed for any depressive symptoms. In addition, respondents who reported at least 1 depressive symptom were posed an additional question as to whether this symptom or symptoms interferes with their daily functioning; we analyze this variable as a binary indicator of whether depressive symptoms affect daily functioning.

Sample size was determined in order to maximize power to detect experimental effects for the primary outcomes of interest (reported separately) [30]. No interim analysis was conducted.

### Statistical analysis

This paper reports on an ITT analysis, focusing on the 80% of individuals randomly sampled at baseline who were invited to participate in the intervention, in conjunction with the sampled individuals in communities assigned to the control arm. Given the intent-to-treat design, all sampled individuals invited to participate in the intervention are included in the analysis, independent of their compliance with their treatment assignment.

The analysis employs logistic regression models fit with generalized estimating equations and including strata fixed effects for district; standard errors are clustered at the level of the village [36]. Odds ratios and 95% confidence intervals are reported for unadjusted and adjusted models. Adjusted models include the following baseline covariates: respondent’s age, respondent’s education level, marriage length, polygamy, socioeconomic status, months between intervention end and endline data collection, and the baseline level of the outcome. Data analysis was conducted in Stata version 14.

## Results

**Fig 1:**
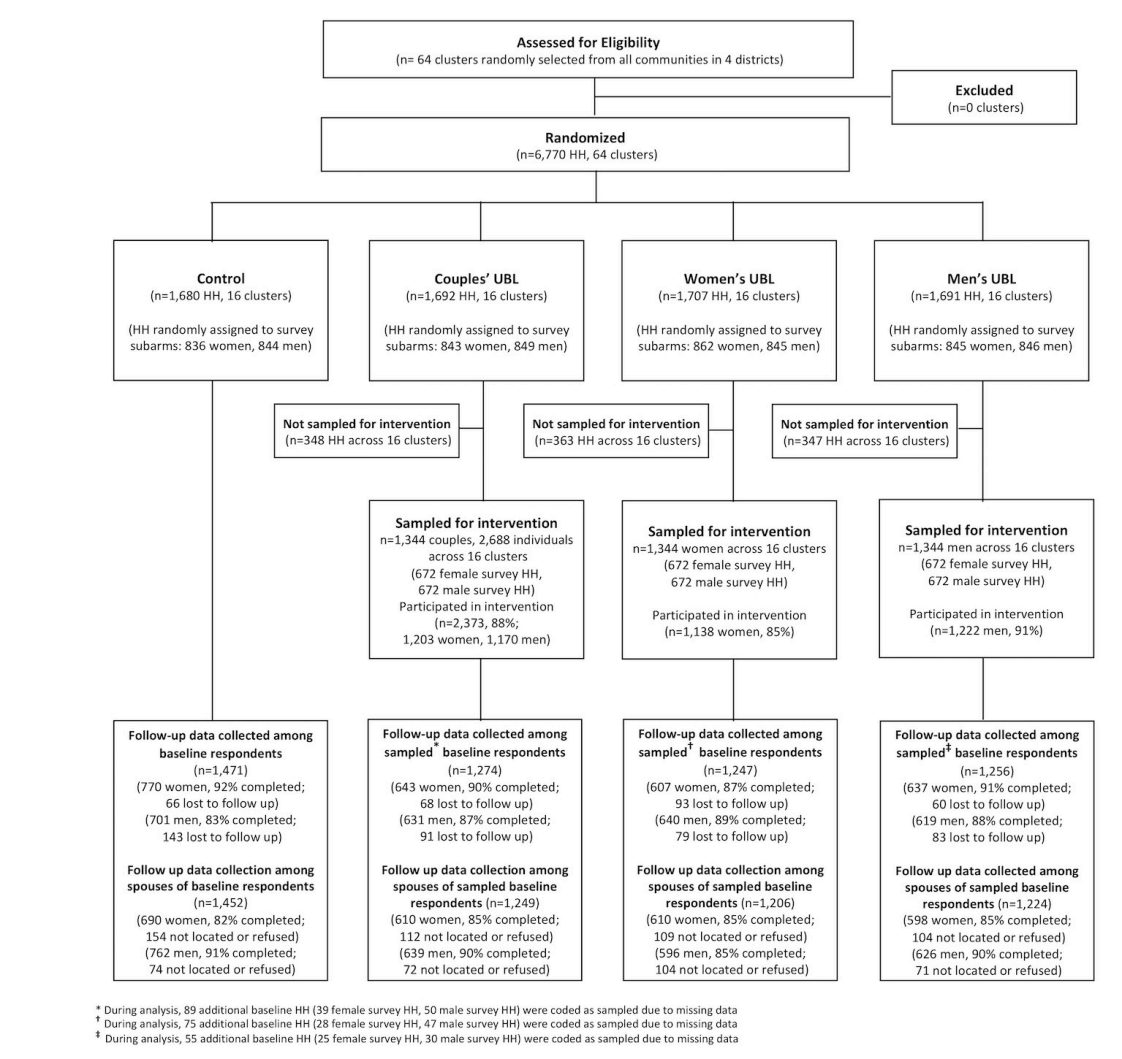
Participant flow diagram. Records around sampling of households for intervention were missing in some intervention communities due to fieldworker error. Any households for which records were missing were coded as sampled for the intervention in order to generate conservative estimates of intervention effects. *During analysis, 89 additional baseline HH (39 female survey HH and 50 male survey HH) were coded as sampled because of missing data. †During analysis, 75 additional baseline HH (28 female survey HH, 47 male survey HH) were coded as sampled due to missing data. ‡ During analysis, 55 additional baseline HH (25 female survey HH, 30 male survey HH) were coded as sampled because of missing data. HH, households; UBL, Unite for a Better Life.

The trial design and sample are summarized in Fig 1. The sample enrolled at baseline includes 6,770 households across 64 clusters; as noted previously, 80% of these households were sampled for inclusion in the intervention, and the analysis focuses on this subsample. Within this subsample, 88% of respondents surveyed at baseline were resurveyed at endline (87% of men and 90% of women), and 87% of spouses of baseline respondents were surveyed at endline (85% of female spouses and 89% of male spouses). No harms were reported.

Baseline characteristics of women and men were broadly similar across evaluation arms, as reported in Table 2. Women were on average 32 years of age, whereas men were 37 years of age. Around 40% of men reported having completed no formal education, and around 75% of women reported no formal education. A total of 61% of households were Muslim.

**Table 2:** Baseline characteristics of women (*N* = 2,944) and men (*N* = 2,987) in the study sample by treatment arm.

	Women				Men			
	Control UBL Arm	Couples' UBL Arm	Women's UBL Arm	Men’s UBL Arm	Control UBL Arm	Couples' UBL Arm	Women’s UBL Arm	Men’s UBL Arm
	(*N* = 836) (%)	(*N* = 711) (%)	(*N* = 700) (%)	(*N* = 697) (%)	(*N* = 844) (%)	(*N* = 722) (%)	(*N* = 719) (%)	(*N* = 702) (%)
Respondent's age category								
<30 years	304 (36.4)	266 (37.4)	249 (35.6)	259 (37.2)	137 (16.2)	144 (19.9)	129 (17.9)	120 (17.1)
30–39	386 (46.2)	308 (43.3)	315 (45.0)	310 (44.5)	350 (41.5)	308 (42.7)	313 (43.5)	281 (40.0)
>39	146 (17.5)	137 (19.3)	136 (19.4)	128 (18.4)	357 (42.3)	270 (37.4)	277 (38.5)	302 (43.0)
Spouse's age category								
<30 years	62 (7.4)	64 (9.0)	59 (8.4)	63 (9.0)	375 (44.4)	344 (47.7)	304 (42.3)	304 (43.3)
30–39	319 (38.2)	258 (36.3)	254 (36.3)	257 (36.9)	315 (37.3)	269 (37.3)	323 (44.9)	294 (41.8)
>39	455 (54.4)	389 (54.7)	387 (55.3)	377 (54.1)	154 (18.3)	109 (15.1)	92 (12.8)	105 (14.9)
Respondent's level of education								
None	641 (76.7)	544 (76.5)	556 (79.4)	535 (76.9)	311 (37.0)	287 (39.8)	278 (38.7)	300 (42.8)
Primary	182 (21.8)	157 (22.1)	139 (19.9)	150 (21.6)	499 (59.3)	397 (55.1)	410 (57.0)	367 (52.4)
Secondary	12 (1.4)	9 (1.3)	5 (0.7)	10 (1.4)	27 (3.2)	28 (3.9)	28 (3.9)	25 (3.6)
Higher	1 (0.1)	1 (0.1)	0 (0.0)	1 (0.1)	4 (0.5)	9 (1.3)	3 (0.4)	9 (1.3)
Spouse's level of education								
None	348 (41.7)	365 (51.3)	341 (48.7)	321 (46.1)	585 (69.5)	464 (64.5)	493 (68.7)	479 (68.2)
Primary	426 (51.1)	308 (43.3)	326 (46.6)	339 (48.6)	240 (28.5)	248 (34.4)	215 (29.9)	214 (30.5)
Secondary	49 (5.9)	29 (4.1)	29 (4.1)	34 (4.9)	14 (1.7)	8 (1.1)	10 (1.4)	9 (1.3)
Higher	11 (1.32)	9 (1.3)	4 (0.6)	3 (0.4)	3 (0.4)	0 (0.0)	0 (0.0)	0 (0.0)
Religion								
Muslim	485 (58.2)	509 (71.7)	400 (57.4)	418 (60.2)	468 (55.7)	518 (72.0)	420 (58.4)	435 (61.9)
Orthodox	279 (33.5)	183 (25.8)	249 (35.7)	202 (29.1)	311 (37.0)	177 (24.6)	233 (32.4)	231 (32.9)
Protestant	57 (6.8)	9 (1.3)	32 (4.6)	66 (9.5)	45 (5.4)	13 (1.8)	49 (6.8)	29 (4.1)
Catholic	12 (1.4)	9 (1.3)	16 (2.3)	8 (1.2)	16 (1.9)	11 (1.5)	17 (2.4)	8 (1.1)
Other	0 (0.0)	0 (0.0)	0 (0.0)	0 (0.0)	1 (0.1)	0 (0.0)	0 (0.0)	0 (0.0)
Substance use								
Frequent past-year drinking (husband/self)	123 (14.6)	102 (14.1)	125 (17.7)	100 (14.1)	160 (19.1)	141 (19.1)	142 (19.2)	125 (17.3)
Reports problems linked to husband/self alcohol use	48 (21.1)	33 (19.3)	53 (26.0)	52 (28.6)	89 (32.4)	46 (21.4)	69 (29.7)	63 (28.9)
Frequent past-year khat use (husband/self)	303 (35.9)	300 (41.6)	248 (35.1)	270 (38.0)	364 (43.4)	382 (51.8)	281 (38.1)	298 (41.4)
Reports problems linked to khat use (husband/self)	40 (9.9)	39 (9.5)	53 (15.8)	39 (11.1)	101 (22.9)	94 (20.4)	109 (29.9)	54 (13.7)
Mental health								
Any depressive symptoms	210 (24.9)	161 (22.3)	157 (22.2)	149 (21.0)				
Symptoms of mild depression	86 (10.2)	88 (12.2)	80 (11.3)	100 (14.1)				
Symptoms of moderate depression	38 (4.5)	30 (4.2)	40 (5.7)	25 (3.5)				
Symptoms of severe depression	0 (0.0)	3 (0.4)	0 (0.0)	0 (0.0)				
Depressive symptoms affecting daily functioning	80 (24.2)	83 (29.4)	71 (25.6)	69 (25.2)				

Table 3 presents summary statistics around engagement in the intervention. Both men and women reported generally high attendance at the 14 intervention sessions; women in the women’s and couples’ UBL arms reported attending on average 11 sessions, and men in the men’s and couples’ UBL arms reported attending 12 sessions on average.

**Table 3:** Engagement in UBL intervention.

	Couples' UBL Arm	Women’s UBL Arm	Men's UBL Arm
	N (%)	N (%)	N (%)
Intervention Engagement–Women’s Reports			
Sessions attended (mean, standard deviation)	11.3	11.2	
	4.6	5.3	
Remembers some or all of what was discussed	535/957	468/813	
	(55.9)	(57.6)	
Shared information	554/853	579/712	
	(64.9)	(81.3)	
Shared information with more than 5 people	331/957	290/813	
	(34.6)	(35.7)	
Continues to meet with UBL group members	768/943	675/791	
	(82.4)	(87.9)	
Satisfaction at high levels	926/948	790/800	
	(97.7)	(98.8)	
Very likely to recommend to a friend	925/945	782/800	
	(97.9)	(97.8)	
Intervention Engagement–Men’s Reports			
Sessions attended (mean, standard deviation)	11.9		12
	3.7		3.4
Remembers some or all of what was discussed	682/985		605/889
	(69.2)		(68.1)
Shared information	845/954		787/844
	(88.6)		(93.2)
Shared information with more than 5 people	267/495		250/443
	(53.9)		(56.4)
Continues to meet with UBL group members	940/980		860/881
	(95.9)		(97.6)
Satisfaction at high levels	892/905		809/820
	(98.6)		(98.7)
Very likely to recommend to a friend	966/973		865/869
	(99.3)		(99.5)

UBL, Unite for a Better Life.

In addition, levels of engagement and social interaction were consistently high for both men and women. Around 50% of all sampled individuals reported that they retained most or all of the material presented in the intervention. The majority of female respondents reported that they had shared information with another individual (65% in the couples’ UBL arm, and 81% in the women’s UBL arm); male respondents were even more likely to report that they had shared information (89% in the couples’ UBL arm, and 93% in the men’s UBL arm). About 35% of female respondents and 55% of male respondents stated they had shared information with more than 5 people, and 85% of female respondents and 95% of male respondents reported continued meetings and discussions with UBL group members around topics related to the program. More than 95% of both male and female respondents reported a high level of satisfaction with the program (greater than or equal to 4 on a 5-point scale) and stated that they would recommend the program to a friend.

Tables 4–6 present summary statistics and estimated treatment effects of UBL on men’s substance use and women’s mental health. In each table, we analyze the prevalence in each intervention arm versus the control arm in an ITT framework and report crude and adjusted odds ratios in conjunction with 95% confidence intervals. Reported levels of past-year substance use at follow-up among men were high according to both men’s and women’s reports but generally higher when reported by men themselves (Table 4, Table 5). Between 10% and 12% of men reported that they were frequently intoxicated with alcohol over the previous year, and between 50% and 60% of men reported that they used khat over the previous year; reported frequency of men’s intoxication according to women was somewhat higher, though reported use of khat according to women was lower. The reported frequency of problems linked to men’s alcohol or khat use ranged between 30% and 50% as reported by both men and women.

**Table 4:** Effects of the UBL intervention on male substance use as reported by women; intention-to-treat analysis.

	Control	Couples	Women's	Men's	Couples' UBL Arm		Women's UBL Arm		Men's UBL Arm	
	*N* (%)	*N* (%)	*N* (%)	*N* (%)	OR	AOR^a^	OR	AOR^a^	OR	AOR^a^
Frequent past-year drinking (husband)	112/763	78/637	107/604	94/635	0.83 (0.46–1.48)	0.74 (0.43–1.27)	1.29 (0.77–2.16)	1.17 (0.71–1.92)	0.98 (0.51–1.90)	0.95 (0.50–1.82)
	(14.7)	(12.2)	(17.7)	(14.8)	*p* = 0.523	*p* = 0.276	*p* = 0.339	*p* = 0.536	*p* = 0.958	*p* = 0.888
Frequent past-year alcohol intoxication (husband)	41/219	27/151	43/209	37/164	1.03 (0.66–1.61)	0.92 (0.56–1.50)	1.13 (0.76–1.70)	1.14 (0.77–1.69)	1.28 (0.76–2.14)	1.22 (0.71–2.09)
	(18.7)	(17.9)	(20.6)	(22.6)	*p* = 0.899	*p* = 0.729	*p* = 0.547	*p* = 0.519	*p* = 0.358	*p* = 0.470
Reports problems linked to husband’s alcohol use	108/217	60/151	109/210	77/162	0.85 (0.48–1.51)	0.91 (0.43–1.93)	1.11 (0.57–2.15)	1.22 (0.53–2.79)	0.81 (0.43–1.54)	0.71 (0.29–1.70)
	(49.8)	(39.7)	(51.9)	(47.5)	*p* = 0.579	*p* = 0.815	*p* = 0.768	*p =* 0.636	*p* = 0.525	*p* = 0.436
Frequent past-year khat use (husband)	234/763	218/638	208/607	219/635	1.18 (0.69–2.04)	1.07 (0.65–1.76)	1.25 (0.69–2.25)	1.29 (0.77–2.18)	1.25 (0.73–2.14)	1.19 (0.72–1.95)
	(30.7)	(34.2)	(34.3)	(34.5)	*p* = 0.542	*p* = 0.775	*p =* 0.463	*p =* 0.336	*p* = 0.424	*p* = 0.496
Reports problems linked to husband's khat use	229/409	256/424	198/336	178/375	1.03 (0.37–2.91)	1.39 (0.27–7.13)	1.24 (0.44–3.47)	1.20 (0.25–5.72)	0.76 (0.30–1.93)	0.57 (0.17–1.90)
	(56.0)	(60.4)	(58.9)	(47.5)	*p* = 0.952	*p* = 0.689	*p* = 0.687	*p* = 0.815	*p* = 0.561	*p* = 0.361

AOR, adjusted odds ratio; OR, odds ratio; UBL, Unite for a Better Life.

^a^Adjusted for respondent’s age, respondent’s schooling category, marriage length, polygamous household, socioeconomic status, whether completed the full or short survey at endline, number of months between end of intervention and endline interview, and the baseline level of the outcome variable

**Table 5:** Effects of the UBL intervention on male substance use as reported by men; intention-to-treat analysis.

	Control	Couples	Women's	Men's	Couples' UBL Arm		Women's UBL Arm		Men's UBL Arm	
	*N* (%)	*N* (%)	*N* (%)	*N* (%)	OR	AOR^a^	OR	AOR^a^	OR	AOR^a^
Frequent past-year drinking	175/701	133/630	149/639	154/619	0.86 (0.47–1.57)	0.87 (0.46–1.61)	0.98 (0.59–1.62)	1.01 (0.60–1.71)	1.21 (0.60–2.44)	1.22 (0.59–2.53)
	(25.0)	(21.1)	(23.3)	(24.9)	*p* = 0.614	*p* = 0.650	*p* = 0.943	*p* = 0.970	*p* = 0.597	*p* = 0.597
Frequent past-year alcohol intoxication	25/241	12/177	27/216	25/206	0.56 (0.36–0.89)	0.57 (0.37–0.89)	1.28 (0.83–1.97)	1.12 (0.70–1.78)	1.21 (0.70–2.10)	1.21 (0.72–2.03)
	(10.4)	(6.8)	(12.5)	(12.1)	*p* = 0.015	*p* = 0.014	*p =* 0.264	*p =* 0.636	*p =* 0.501	*p* = 0.474
Reports problems linked to alcohol use	88/240	82/177	94/215	93/206	1.41 (0.92–2.15)	1.44 (0.93–2.23)	1.37 (0.93–2.03)	1.40 (0.91–2.17)	1.41 (0.92–2.17)	1.42 (0.93–2.17)
	(36.7)	(46.3)	(43.7)	(45.1)	*p* = 0.115	*p* = 0.102	*p* = 0.114	*p* = 0.127	*p* = 0.118	*p* = 0.102
Frequent past-year khat use	375/701	426/631	340/640	354/618	2.86 (1.58–5.20)	3.14 (1.70–5.81)	1.12 (0.60–2.09)	1.13 (0.60–2.12)	1.43 (0.68–3.00)	1.38 (0.67–2.85)
	(53.5)	(67.5)	(53.1)	(57.3)	*p* = 0.001	*p* < 0.001	*p* = 0.713	*p* = 0.701	*p* = 0.346	*p* = 0.378
Reports problems linked to khat use	196/403	200/455	143/367	160/398	0.89 (0.48–1.66)	0.86 (0.46–1.61)	0.77 (0.43–1.36)	0.75 (0.42–1.34)	0.84 (0.48–1.49)	0.84 (0.48–1.46)
	(48.6)	(44.0)	(39.0)	(40.2)	*p* = 0.719	*p* = 0.643	*p* = 0.391	*p* = 0.325	*p* = 0.488	*p* = 0.535

AOR, adjusted odds ratio; OR, odds ratio; UBL, Unite for a Better Life.

^a^Adjusted for respondent’s age, respondent’s schooling category, marriage length, polygamous household, socioeconomic status, whether completed the full or short survey at endline, number of months between end of intervention and endline interview, and the baseline level of the outcome variable.

**Table 6:** Effects of the UBL intervention on women’s mental health; intention-to-treat analysis.

	Control	Couples	Women's	Men's	Couples' UBL Arm		Women's UBL Arm		Men's UBL Arm	
	*N* (%)	*N* (%)	*N* (%)	*N* (%)	OR	AOR^a^	OR	AOR^a^	OR	AOR^a^
Any depressive symptoms	123/770	124/643	106/637	112/637	1.28 (0.93–1.77)	1.26 (0.91–1.76)	1.12 (0.82–1.53)	1.11 (0.82–1.52)	1.13 (0.82–1.55)	1.13 (0.83–1.53)
	(16.0)	(19.3)	(17.5)	(17.6)	*p* = 0.134	*p* = 0.171	*p* = 0.472	*p* = 0.493	*p* = 0.464	*p* = 0.445
Symptoms of mild depression	86/770	82/643	62/637	74/637	1.17 (0.81–1.70)	1.14 (0.80–1.63)	0.91 (0.65–1.26)	0.88 (0.63–1.23)	1.05 (0.72–1.53)	0.99 (0.70–1.40)
	(11.2)	(12.8)	(10.2)	(11.6)	*p* = 0.390	*p* = 0.474	*p* = 0.560	*p* = 0.466	*p* = 0.810	*p* = 0.944
Symptoms of moderate depression	33/770	37/643	42/637	34/637	1.38 (0.91–2.09)	1.35 (0.90–2.04)	1.66 (1.11–2.49)	1.59 (1.10–2.30)	1.26 (0.83–1.93)	1.30 (0.86–1.97)
	(4.3)	(5.8)	(6.9)	(5.3)	*p* = 0.127	*p* = 0.152	*p* = 0.013	*p* = 0.015	*p* = 0.278	*p* = 0.209
Symptoms of severe depression	4/770	5/643	2/637	4/637	1.53 (0.46–5.12)	1.56 (0.41–5.94)	0.61 (0.12–3.13)	0.66 (0.13–3.44)	1.21 (0.34–4.32)	1.59 (0.45–5.67)
	(0.5)	(0.8)	(0.3)	(0.6)	*p* = 0.487	*p* = 0.516	*p* = 0.557	*p* = 0.623	*p* = 0.764	*p* = 0.474
Depressive symptoms affecting daily functioning	185/331	176/311	170/307	169/307	1.01 (0.68–1.51)	1.28 (0.85–1.92)	1.03 (0.64–1.66)	1.18 (0.67–2.09)	0.95 (0.59–1.51)	0.91 (0.56–1.49)
	(55.9)	(56.6)	(58.0)	(55.0)	*p* = 0.944	*p* = 0.239	*p* = 0.903	*p* = 0.568	*p* = 0.816	*p* = 0.715

AOR, adjusted odds ratios; OR, odds ratio; UBL, Unite for a Better Life.

^a^Adjusted for respondent’s age, respondent’s schooling category, marriage length, polygamous household, socioeconomic status, whether completed the full or short survey at endline, number of months between end of intervention and endline interview, and the baseline level of the outcome variable.

Depression daily functioning is reported for all those who have any depressive symptoms, but missing for some individuals (11 in control, 9 in female, male, and couples each).

The estimated treatment effects using women’s reports suggest that the intervention did not generate any significant shifts in substance use (Table 4). The table presents both unadjusted and adjusted estimates; the latter estimates are adjusted for baseline covariates including age, education level, marriage length, polygamy, socioeconomic status, months between intervention and endline data collection, and the baseline level of the outcome variable. There is no evidence of any shift in frequent past-year spousal drinking (couples’ UBL arm adjusted odds ratio [AOR] = 0.74, CI 0.43–1.27, *p* = 0.276; women’s UBL arm AOR = 1.17, CI 0.71–1.92, *p* = 0.536; men’s UBL arm AOR = 0.95, CI 0.50–1.82, *p* = 0.888), reported problems linked to spousal alcohol use (couples’ UBL arm AOR = 0.91, CI 0.43–1.93, *p* = 0.815; women’s UBL arm AOR = 1.22, CI 0.53–2.79, *p* = 0.636; men’s UBL arm AOR = 0.71, CI 0.29–1.70, *p* = 0.436), frequent past-year spousal khat use (couples’ UBL arm AOR = 1.07, CI 0.65–1.76, *p* = 0.775; women’s UBL arm AOR = 1.29, CI 0.77–2.18, *p*-value = 0.336; men’s UBL arm AOR = 1.19, CI 0.72–1.95, *p* = 0.496), or reported problems linked to spousal khat use (couples’ UBL arm AOR = 1.39, CI 0.27–7.13, *p* = 0.689; women’s UBL arm AOR = 1.20, CI = 0.25–5.72, *p* = 0.815; men’s UBL arm AOR = 0.57, CI 0.17–1.90, *p* = 0.361).

The estimated treatment effects using men’s reports are reported in Table 5; again, both unadjusted and adjusted estimates are reported. There was a statistically significant decline in the probability of reporting frequent past-year intoxication (AOR = 0.57, 95% CI 0.37–0.89, *p* = 0.014) and a statistically significant increase in the probability of reporting frequent past-year khat use (AOR = 3.14, 95% CI 1.70–5.81, *p* < 0.001), both in the couples’ UBL arm (Table 5). There is no evidence of any shift in frequent past-year drinking (couples’ UBL arm AOR = 0.87, CI 0.46–1.61, *p* = 0.650; women’s UBL arm AOR = 1.01, CI 0.60–1.71, *p* = 0.970; men’s UBL arm AOR = 1.22, CI 0.59–2.53, *p* = 0.597), no shift in reported problems linked to alcohol use (couples’ UBL arm AOR = 1.44, CI 0.93–2.23, *p* = 0.102; women’s UBL arm AOR = 1.40, CI 0.91–2.17, *p* = 0.127; men’s UBL arm AOR 1.42, CI 0.93–2.17, *p* = 0.102), no shift in reported past-year khat use in the women’s or men’s arm (women’s UBL arm AOR 1.13, CI 0.60–2.12, *p* = 0.701; men’s UBL arm AOR = 1.38, CI 0.67–2.85, *p* = 0.378), and no shift in reported problems linked to khat use (couples’ UBL arm AOR = 0.86, CI 0.46–1.61, *p* = 0.643; men’s UBL arm AOR = 0.75, CI 0.42–1.34, *p* = 0.325; women’s UBL arm AOR = 0.84, CI 0.48–1.46, *p* = 0.535).

For reported depressive symptoms among women, at follow-up, the reported prevalence of symptoms of mild depression among women was between 10% and 12%; the prevalence of symptoms of moderate depression was between 5% and 7%, and symptoms of severe depression were very rare (less than 1%). Among women who reported at least one depressive symptom, however, between 50% and 60% reported that this symptom interfered with their daily functioning (Table 6).

The estimated coefficients suggest there was a large and statistically significant increase in reported symptoms of moderate depression in the women’s UBL arm only (AOR = 1.54, 95% CI 1.13–2.41, *p* = 0.010). However, there was no evidence of any changes in any reported depressive symptoms (couples’ UBL arm AOR = 1.26, CI 0.91–1.76, *p* = 0.171; women’s UBL arm AOR = 1.11, CI 0.82–1.52, *p* = 0.493; men’s UBL arm AOR = 1.13, CI 0.83–1.53, *p* = 0.445), reported symptoms of mild depression (couples’ UBL arm AOR = 1.14, CI 0.80–1.63, *p* = 0.474; women’s UBL arm AOR = 0.88, CI 0.63–1.23, *p* = 0.466; men’s UBL arm AOR = 0.99, CI 0.70–1.40, *p* = 0.944) reported symptoms of moderate depression in the couples’ and men’s UBL arms (couples’ arm AOR = 1.35, CI 0.90–2.04, *p* = 0.152; men’s UBL arm AOR = 1.30, CI 0.86–1.97, *p* = 0.209), or reported symptoms of severe depression or the probability that depressive symptoms affected daily functioning (as reported in Table 6).

## Discussion

This analysis suggests that the UBL intervention when delivered to couples is associated with a significant reduction in frequent past-year alcohol use by men at 24 months’ follow-up. More specifically, men in the couples’ UBL arm reported a significant reduction in the probability that they were frequently intoxicated with alcohol in the last year compared with men in the control arm. However, there was an increase in reported frequent past-year khat use for men in this arm. There was no shift in reported use of substances by men in the women’s UBL or men’s UBL arms, or in women’s reports of their spouses’ substance use in any arm.

With respect to mental health outcomes, there is no evidence that the UBL intervention was associated with reductions in the prevalence of depressive symptoms in any treatment arm. Interestingly, there was an increase in symptoms of moderate depression reported in the women’s UBL arm, but no evidence of any change in symptoms of mild or severe depression and no shift in the effect of depressive symptoms on daily functioning in any of the arms. As explored further next, the shift in reported symptoms of moderate depression likely represents changes in patterns of reporting or disclosure.

Relative to the literature, the results around substance use are broadly consistent with the evidence presented in the evaluation of Stepping Stones as well as Stepping Stones in conjunction with Creating Futures, interventions seeking to enhance sexual health among youth in South Africa; the programs generated a reduction in alcohol misuse among men [17,18]. In this trial, there was a reduction in frequent alcohol intoxication among men and a concomitant increase in frequent khat use. Khat use is not measured or reported in the South African trials given that it is not relevant in that context; the original Stepping Stones trial does analyze use of other drugs, and there is no evidence of any significant effects. Notably, in this trial, the observed shifts in alcohol and khat use are observed only in the couples’ UBL arm, despite the fact that knowledge around the consequences of alcohol use was identified as an intermediate outcome of interest for the men’s intervention. This pattern suggests that men may be more sensitive to discussions around the consequences of substance use when those discussions include their spouses.

Other recent papers analyzing IPV interventions targeting women, men, or couples in South Africa, Côte d’Ivoire, and Uganda do not measure or report effects on substance use [37–40]. A recent intervention targeting male engagement in reproductive and maternal health and violence prevention in Rwanda included an intervention session focused on risks and consequences of alcohol and drug use, but the trial did not measure experimental effects along these dimensions [41].

The results around mental health differ from previous research in that there is some evidence that the intervention was associated with an increase in reported depressive symptoms when delivered to women. In previous papers, Stepping Stones had no effect on depression among women, and only the intervention implemented in conjunction with Creating Features showed effectiveness in reducing depression among men [17,18]; other recent work from Ghana evaluating a rapid response system seeking to prevent IPV reported a decrease in depression among women [29]. The different pattern observed in this paper could reflect 2 potential channels.

First, this could be a reporting effect: Women who participated in UBL may now be more likely to report depressive symptoms. Previous studies have presented evidence of under-reporting of depression symptoms by women [42], linked to several pathways: Women may not be aware of symptoms of depression [43], they may minimize or deny the symptoms [44], or they may be reluctant to disclose [43]. It is plausible that the intensive group sessions implemented as part of UBL addressed some of these challenges. The main trial findings provide further support to this hypothesis, as there were significant increases in spousal communication around sexuality and HIV in the intervention arms, demonstrating increased comfort discussing sensitive topics [30].

Second, the increase in symptoms of moderate depression could reflect changes in actual symptoms as women became more aware of the frequency of IPV and its consequences, without any concomitant reduction in the frequency of violence in the women’s UBL arm [30]. Recent evidence in Bangladesh found that women’s justification of IPV was associated with fewer mental health symptoms; the authors suggest that adherence to these social norms allow women to rationalize their experience of IPV, limiting the mental health consequences [45]. Insofar as the UBL intervention shifted social norms around IPV, this may have generated an increase in symptoms of depression. However, the absence of any shift in symptoms of mild or severe depression or challenges linked to depression suggests that the reporting channel may be more meaningful. In addition, the UBL intervention may have provided women with tools to manage challenges around depressive symptoms: In particular, additional social interaction accessed through the intervention. Two key points of evidence are consistent with an increase in social support and social cohesion: Both male and female participants shared information with other individuals outside of the program, and 85% of female and 95% of male respondents reported continuing to meet with members of their UBL group.

This evaluation has a number of strengths. To our knowledge, this is one of the first RCTs to report substance use and mental health outcomes in the context of an intervention targeting IPV and risky sexual behavior. These outcomes were not specified as primary or secondary outcomes in the trial registry; however, they were specified in the original full protocol. The only evaluations reporting parallel effects are 2 related evaluations of the Stepping Stones intervention in South Africa [17,18]. This is also the first RCT to analyze the differential effects of delivering a gender-transformative IPV prevention intervention to women, men, and couples on the outcomes of interest, again, with the caveat that these were not primary outcomes. In addition, the evaluation sample is representative of the setting given that households were randomly selected for inclusion, and loss to follow-up was minimal.

Our trial has several limitations. First, we used a short 5-question module about substance use rather than a more detailed scale capturing alcohol use (such as the Alcohol Use Disorders Identification Test [AUDIT] scale); the AUDIT scale has been widely used to measure alcohol consumption, including in Ethiopia, though it has not been validated in this context [46–48]. We also did not measure substance use among women despite the fact that published Demographic and Health Survey UBL (DHS) data suggest rates of alcohol use among women of reproductive age in Ethiopia is nontrivial [31]. Previously reported rates of khat use are significantly lower among women compared with men, though reported use of both substances by women may be meaningfully affected by social desirability bias [31]. Existing literature has highlighted that substance use among women may be an adverse effect of exposure to IPV, but the trial did not evaluate shifts in substance use among women [2].

Second, we rely on self-reported data for substance use. In particular, men’s reporting of substance use may be subject to social desirability bias given that the intervention highlighted adverse consequences of substance use, and one indicator of interest relies on self-reported alcohol intoxication (literally described as drunkenness), a term that may be pejorative. In addition, no significant effects of the intervention are observed on men’s substance use as reported by women. Accordingly, one interpretation of the observed pattern is that the observed decline in men’s use of alcohol in the couples’ UBL arm reflects social desirability bias in reporting, particularly given that the shift in reported frequent alcohol intoxication could reflect changes in the respondent’s own perception of this state. This is a meaningful challenge in the interpretation of the results. However, 2 points suggest that the observed pattern does not solely reflect social desirability bias. It seems surprising that male respondents providing socially desirable responses would report an increase in khat use, given that the intervention generally also highlighted adverse effects of khat (albeit with less emphasis than in the case of alcohol). It is more plausible that they would similarly report lower levels of khat use or at least consistent levels. In addition, male respondents in the men’s UBL arm—who were similarly directly exposed to intervention programming around substance use—did not report any behavioral changes along this dimension.

Third, we rely on the PHQ-9 instrument to measure depressive symptoms among women. The PHQ-9 questionnaire has been validated for use in Ethiopia [34], but there are few published estimates of the prevalence of depression or depressive symptoms in similar samples. Evidence from a population of adults in northwest Ethiopia suggests a prevalence of 17.5% for any depressive symptoms measured using the PHQ-9, broadly consistent with what is observed in this sample [49]. A separate paper reports a prevalence rate for depressive symptoms of 29.5% among a population of pregnant women in Sodo district, also measured using the PHQ-9; these rates are somewhat higher than the rates observed in this sample, but that may reflect primarily challenges linked to childbearing [50]. A third paper reports an estimated prevalence rate of 4.8% for depressive episodes in a sample of women of reproductive age in the same region, measured using the Composite International Diagnostic Interview (CIDI) [31]. Other recent papers estimate a prevalence of depressive episodes among women of 9.5% in a nationwide sample [51] and 8.7% for women in southwest Ethiopia [52]. A meta-analysis of the prevalence of depression in Ethiopia reports that the average prevalence of depression is 11% (pooled across men and women) [53], again, broadly consistent with what is observed in this sample.

Although the available evidence suggests the PHQ-9 instrument performs well in this setting, there may be persistent measurement challenges, particularly given that the population of interest is characterized by low levels of literacy. Measuring symptoms of severe depression may be particularly difficult. Existing literature has highlighted challenges in establishing cultural relevance for brief psychiatric screening instruments in sub-Saharan Africa, and this remains an important direction for research [54]. In addition, we did not measure additional mental health symptoms that may be linked with IPV (e.g., anxiety and stress), despite the potential for the intervention to address these related symptoms.

Our findings have implications for future research, programming, and policy. First, the summary statistics around substance use as reported by both men and women highlight interesting patterns that have implications for future programming targeting substance use and associated risky behaviors in this region. Women in general report lower prevalence rates of frequent alcohol and khat use and frequent alcohol intoxication among their spouses relative to men’s reports, suggesting that some substance use may be concealed from women. Recent literature has documented that although concordance in self-reports and spousal reports of alcohol use is relatively high in developed country settings, in sub-Saharan Africa, concordance may be significantly lower; in Zambia, women reported significantly higher rates of alcohol use by their spouses relative to their spouses’ self-reports [55]. In this context, the pattern appears to be inverted (women report lower rates of alcohol and khat use by their spouses, relative to self-reports), a pattern that could be explored further in future research.

In addition, in this setting, frequent use of khat is much more common than frequent use of alcohol among men. In the pooled sample, 10% of men reported they were frequently intoxicated with alcohol, whereas nearly 58% reported they frequently used khat. Similarly, 20% of women reported their spouses were frequently intoxicated, and 33% reported that their spouses frequently used khat. The prevalence of frequent khat use and the existing evidence that khat use may be associated with IPV [10–16] suggests the importance of using culturally informed strategies to address this challenge in public health programming, focusing on addressing potential harms from khat use while noting its cultural and religious significance [9]. In addition, the evidence of shifts in alcohol and khat use in opposite directions in the couples’ arm suggests that future interventions may benefit from increased exploration of substance substitution, a recognized challenge in which individuals prompted to address patterns of substance use replace the substance identified as problematic with a different substance perceived as “safe” [56–58]. Existing literature recommends substance substitution be more extensively assessed in research related to addiction [59], and our findings support this guidance.

In the area of mental health, our findings indicate that the UBL intervention did not effectively reduce depressive symptoms among the female participants, even in the men’s arm in which IPV declined. An implication for future programming is that similar interventions should explore the inclusion of additional content linked to coping with stress and trauma linked to IPV. For example, a trial currently underway in Congolese refugee camps in Tanzania seeks to evaluate an IPV intervention that integrates advocacy and structured psychosocial support; the authors argue that integrating this psychosocial support may render the preventive intervention more effective, vis-à-vis an intervention that focuses solely on advocacy [60]. Other recent literature has highlighted the limited evidence base around the effects of mental health interventions on the prevention and reduction of IPV [61], suggesting the potential importance of further exploring the role of targeted interventions addressing the nexus between IPV and mental health.

This trial presents new evidence around the impact of a 14-session gender-transformative intervention on depressive symptoms among women and substance use by men. The additional findings from this trial make a unique contribution to the existing literature by revealing new relationships between a 14-session gender*-*transformative intervention for IPV and depressive symptoms among women and substance use by men. The evidence presented here highlights that further research may be useful in identifying how IPV prevention interventions can be optimized to target not only IPV but also substance use that may be correlated with violence used by perpetrators, as well as mental health challenges and trauma experienced by women who are survivors.

## Supporting information

S1 CONSORT checklist CONSORT, Consolidated Standards of Reporting Trials. (DOCX)

S1 Text Trial protocol. (DOC)

S2 Text Pre-analysis plan. (PDF)

## Abbreviations

AOR: adjusted odds ratio
AUDIT: Alcohol Use Disorders Identification Test
CIDI: Composite International Diagnostic Interview
DHS: Demographic and Health Surveys
EPHA: Ethiopian Public Health Association
HAPCO: HIV/AIDS Prevention and Control Office
IPV: intimate partner violence
ITT: intention-to-treat
PHQ-9: Patient Health Questionnaire
RCT: randomized controlled trial
RHANI: Raising HIV Awareness in Non-HIV-Infected Indian Wives
UBL: Unite for a Better Life
