# Supplementary material for: An intimate partner violence prevention intervention for men, women, and couples in Ethiopia: Additional findings on substance use and depressive symptoms from a cluster-randomized controlled trial
Source: PLoS Med. 2020 Aug 18;17(8):e1003131. doi: 10.1371/journal.pmed.1003131 (PMC7433854; doi:10.1371/journal.pmed.1003131)
Supplement: S1 Text — (DOC) [file pmed.1003131.s002.doc]

|  | Massachusetts Institute of TechnologyCommittee on the Use ofHumans as Experimental Subjects | Application # (assigned by  COUHES) | 1211  005333 |
| --- | --- | --- | --- |
|  |  | **Date** | **UPDATED Aug. 2019** |

**Application for Approval to Use Humans as Experimental Subjects (Standard Form)**

*Please answer every question. Positive answers should be amplified with details. You must mark N/A where the question does not pertain to your application. Any incomplete application will be rejected and returned for completion.* ***A completed CHECKLIST FOR STANDARD APPLICATION FORM must accompany this application.***

### BASIC INformation

| 1. Title of Study | | | | | |
| --- | --- | --- | --- | --- | --- |
| Using Cultural Ceremonies to Reduce Intimate Partner Violence and HIV Transmission | | | | | |
| 2. Principal Investigator | | | | | |
| Name: Dr. Vandana Sharma | | Building and Room #: E19-201 | | | |
| Title: Research Scientist | | Email: vsharma@povertyactionlab.org | | | |
| Department: J-PAL | | Phone: 443-703-8457 | | | |
| **3. Study Personnel** | | | | | |
| *All* key personnel[[1]](#footnote-2) **including the PI** *must be listed below, with a brief statement of qualifications and study role(s).*  ***Important Note:*** *all key personnel are required to complete Human Subject training before work begins on the project.* | | | | | |
| *Investigators and other personnel [and institution(s)]*  *include email address:* | *Qualifications: Describe briefly* | | | *Study role(s): (Check box to the right if person will be obtaining consent.)* |  |
| **Dr. Vandana Sharma**  vsharma@povertyactionlab.org | **MD, MPH (J-PAL)** | | | **PI** |  |
| **Jessica Leight**  j.leight@cgiar.org | **Research Fellow, IFPRI** | | | **Co-Investigator** |  |
| **Dr. Negussie Deyessa**  [negussie.deyessa@psychiat.umu.se](mailto:negussie.deyessa@psychiat.umu.se) | **Professor, Addis Ababa University** | | | **Co-Investigator** |  |
| **Samuel Tewolde**  [mysamuel11@yahoo.com](mailto:mysamuel11@yahoo.com) | **Independent consultant** | | | **Lead qualitative researcher** |  |
| **Arsena Solomon**  [arsemasol@gmail.com](mailto:arsemasol@gmail.com) | **Independent consultant** | | | **Lead qualitative researcher** |  |
| **4. Collaborating Institutions.** *If you are collaborating with another institution(s) then you must obtain approval from that institution’s institutional review board, and forward copies of the approval to COUHES)* | | | | | |
| Addis Ababa University - letter of cooperation (IRB in process)  Ethiopian Public Health Association- letter of cooperation (no IRB)  EngenderHealth- letter of cooperation (cede to MIT’s authority)  American University ceded to MIT in 2017 | | | | | |
| **5. Location of Research.** *If at MIT please indicate where on campus. If you plan to use the facilities of the Clinical Research Center you will need to obtain approval of the MIT Catalyst Clinical Research Center.* | | | | | |
| Ethiopia | | | | | |
| 6. **Funding**. *If the research is funded by an outside sponsor, please enclose one copy of the research proposal with your application. A draft of the research proposal is acceptable.* ***Do not leave this section blank. If your project is not funded check No Funding.*** | | | | | |
| 1. Sponsored Project Funding: | | | | | |
| Current Proposal Proposal # **___**________________  Sponsor **__**__________________________________________________  Title **_**______________________________________________________  Current Award Account # **3978733**____________________  Sponsor **Anonymous Foundation**_________________________________________  Title **Using Cultural Ceremonies to Reduce Intimate Partner Violence and HIV Transmission** ______________________________________ | | | | | |
| 1. Institutional Funding: | | | | | |
| Gift  Departmental Resources  Other (explain) **__**______________________________________  No Funding | | | | | |
| **7. Statement of Financial Interest** | | | | | |
| Does the principal investigator or any key personnel involved in the study have any financial interest in the research?  Yes No  If yes then attach a **Supplement for Disclosure of Financial Interest** for each individual with an interest. *This supplement, together with detailed guidance on this subject and definitions of the highlighted terms, is available on the COUHES web.* | | | | | |
| **8. Human Subjects Training**. *All study* personnel **MUST** *take and pass a training course on human subjects research. MIT has a web-based course that can be accessed from the main menu of the COUHES web site. COUHES may accept proof of training from some other institutions. List the names of all study personnel and indicate if they have taken a human subjects training course.* | | | | | |
| Vandana Sharma (certificate attached)  Jessica Leight (certificate attached)  Negussie Deyessa (certificate attached) | | |  | | |
| 9. Anticipated Dates of Research | | | | | |
| Start Date: February 1, 2013 | | | Completion Date: March 1, 2022 | | |

1. STUDY INFORMATION

| **1. Purpose of Study.** *Please provide a concise statement of the background, nature and reasons for the proposed study. Use non-technical language that can be understood by non-scientist members of COUHES.* |
| --- |
| Violence against women (VAW), the most extreme manifestation of the unequal power balance between women and men is a ubiquitous and pervasive global public health concern. In a study of 10 countries, the lifetime prevalence of physical and/or sexual intimate partner violence (IPV), one of the most common forms of VAW, was found to be between 15 and 71% (1). There is growing evidence that IPV has a variety of negative health consequences and is linked to HIV risk. For example, one study found that women who experienced IPV had a 56% excess risk of HIV infection than those who had not (2). Culturally appropriate, community-based interventions that stimulate behavioral change have the potential to be an extremely valuable weapon to address IPV and in turn improve health outcomes and potentially reduce transmission of HIV/AIDS. However, few such interventions have been rigorously evaluated.  The primary objective of the study is to rigorously evaluate using a randomized controlled trial a community-based intervention focused on IPV and HIV delivered in the context of the Ethiopian coffee ceremony, a culturally established forum for community discussion and conflict resolution. By linking the intervention to a traditional cultural ceremony, it may be more culturally acceptable and potentially more effective in altering community and social norms and behaviors. The intervention will involve fourteen 2-hour sessions led by a trained facilitator with the aim of changing community norms around gender and sexuality and building stronger, healthier relationships. One hundred and twenty eight villages in four districts in Ethiopia will be randomly assigned to one of 4 study arms (3 treatment and 1 control group). The intervention will be provided to women only (Arm 1), men only (Arm 2), or to both men and women (Arm 3). Women and men in the control group will receive a short educational session focused on violence reduction.  The study will take place over a period of 3 years and will assess the impact of the intervention on women’s past year risk of physical and/or sexual violence, and male perpetration of violence and on HIV knowledge, attitudes and behaviors. Data will be collected at baseline and at 18 months after baseline. This project, a collaboration between the Abdul Latif Jameel Poverty Action Lab, Addis Ababa University, the Ethiopian Public Health Association, and EngenderHealth, is an innovative and culturally appropriate approach targeting two critical public health issues. The results of this study will add to the limited evidence base on strategies which can be effectively used to combat IPV and in turn HIV/AIDS, as well as the mechanisms through which these programs have an effect. If proven effective, the intervention could be adapted to link with other cultural or religious ceremonies or processes in other parts of Africa. |
| **2. Study Protocol.** *For* ***biomedical, engineering and related research****, please provide an outline of the actual experiments to be performed. Where applicable, provide a detailed description of the experimental devices or procedures to be used, detailed information on the exact dosages of drugs or chemicals to be used, total quantity of blood samples to be used, and descriptions of special diets.*  For applications in the **social sciences, management and other non-biomedical disciplines** please provide a detailed description of your proposed study. Where applicable, include copies of any questionnaires or standardized tests you plan to incorporate into your study. If your study involves interviews please submit an outline indicating the types of questions you will include.  *You should provide sufficient information for effective review by non-scientist members of COUHES. Define all abbreviations and use simple words. Unless justification is provided this part of the application must not exceed 5 pages.*  *Attaching sections of a grant application is not an acceptable substitute.* |
| The proposed evaluation is a cluster randomized controlled trial, with randomization at the village level. As the intervention involves groups, individual level randomization would not be appropriate. Villages will be randomly assigned to one of 4 study arms (3 intervention and 1 control arm): 1) Women only participate in the intervention, 2) Men only participate, 3) Both men and women (couples) participate, 4) Women and men receive the control intervention of a short informational session on violence reduction.   1. **Intervention**   The Ethiopian traditional coffee ceremony will be used as an entry point for a community-based intervention to provide information, change behavior around IPV and improve gender equity and intra-couple relations. The Ethiopia coffee ceremony was chosen because it is a culturally established forum for community discussion and conflict resolution and an integral part of Ethiopian life. The intervention will involve weekly coffee ceremonies, during which approximately 20 members of the community will participate in education and discussions centered on gender issues, sexuality, communication and conflict resolution, HIV/AIDS and its link with violence, as well as HIV/AIDS prevention. Each coffee ceremony will be moderated by a female or male facilitator trained in participatory learning, moderation, HIV/AIDS prevention, counseling, and gender issues. Female facilitators will moderate the women only groups, while male facilitators will lead the men only or couples groups. Facilitators will also be trained in the safety and ethics of conducting interventions on violence, minimizing risk to participants and ensuring their safety. The intervention will involve one 2-hour session per group each week for a total of 14 weeks. EngenderHealth, an international organization with strong expertise in transforming harmful gender norms, will develop and pretest the full curricula for each intervention arm and will also develop and implement the training of trainers. The Addis Ababa University School of Public Health will be responsible for implementing the intervention.   1. **Treatment arms**   In the first study arm, the intervention will be provided to women in the participating villages; in the second study arm, it will be provided to men; and in the third arm, it will be provided to mixed gender groups in which couples attend together. Women and men in the control arm will receive a short educational session focused on violence reduction.   1. **Pilot study**   A small pilot was already conducted in 2012 (COUHES # 12010048) to test the feasibility of delivering the intervention within the traditional coffee ceremony, and to assess acceptability of the messages and group composition. During that pilot, there was a very strong turnout, and high levels of participation even in the mixed gender group. The main feedback provided by participants was the need for more sessions.  A new pilot study will be conducted once the full 14 session curriculum has been developed, prior to launching the full-fledged trial, to measure attendance and evaluate methods for minimizing attrition over the full course of the 14-week intervention and survey with the aim of improving program implementation and data collection. The full 14-week intervention will be piloted with one group of participants in each study arm (1 group of women, 1 group of men and 1 group of couples) using the **“Pilot Questionnaire for Female Respondents”** submitted to the IRB in November 2012. This pilot questionnaire will be used to develop the female and male baseline questionnaires.  In order to minimize attrition, the intervention will include a small in-kind incentive for attendance of a health-promoting good valued approximately $0.50 such as soap, painkiller, or children’s shoes. The incentive will be provided to all participants having attended the full complement of sessions at the 7-week mark, as well as at the completion of the intervention. Certificates will be awarded to participants completing all 14 sessions. During each session, participation will be recorded and at the end of the 14-week cycle attrition will be measured in each group; the target attrition rate is no higher than 75%. Structured interviews will also be conducted with participants to obtain feedback about the intervention.  During the second stage of piloting, the full intervention will be administered to six separate groups with two groups in each study arm. Adjustments will be made based on participant feedback and attrition rates during the first stage. If the attrition rate was higher than desired during stage 1, then incentives during stage 2 will be increased in value and potentially in frequency. At the conclusion of stage 2, the endline survey will be administered to all participants enrolled in this intervention cycle, irrespective of attendance. To facilitate this process, tracking information (name, location of home and cell phone number) will be collected from all participants upon enrollment in the intervention. The target attrition rate for the survey is 90%, balanced across participants who did and did not complete the intervention.  The pilot study will also involve development and testing of the data collection tools. Questions on the acceptability and attitudes towards interviewing both men and women in the same household about violence will be included.   1. **Target population and sampling plan**   The study will be undertaken in Meska, Mareko, Sedo, and Silte districts, located in the Gurague Zone in the Southern Nations and Nationalities and Peoples Regional State in Ethiopia. Women and men fulfilling the following inclusion criteria, and their partners if applicable, will be selected to participate in the evaluation: married or cohabitating individuals aged 18 to 49 years, and resident in rural kebeles in the study districts for at least the last six months.  Using the list of villages within the peasant associations of the four districts as a sampling frame, 64 villages will be selected using random sampling. Households in the 64 villages will be mapped during a partial census and GPS information will be recorded. For participation in the intervention, baseline and post-intervention surveys, 106 households in each village will be selected using simple random sampling.  In the women only, couple and control arms, one woman will be selected by lottery method from all eligible women within the household. This woman (and her husband, if applicable) will participate in the intervention or control information session, and either the wife or her husband will be interviewed at baseline and 18 months post-baseline. In the men only study arm, the male household head will participate in the intervention, and either he or his wife will participate in the survey.  For data collection, households in the village will be randomized to a male survey group (53 households) and a female survey group (53 households). In the female survey groups, the woman selected for the intervention or the wife of the man selected for the intervention will be interviewed during baseline, whereas in the male survey group, the man selected to participate in the intervention or the husband of the woman selected will be interviewed.  At endline, a survey will be administered to all baseline respondents. In addition, a shorter survey will be administered to their spouses.   1. **Statistical Power and sample size**   The power calculations for this study follow the methodology provided in the standard review article by Hayes and Bennett (19). Sample size was determined using the formula for two proportion population for 5 key indicators that the intervention is designed to impact: the prevalence of physical violence by an intimate partner in the last 12 months (1), the prevalence of sexual violence by an intimate partner in the last 12 months (1), the prevalence of male perpetration of violence in the last 12 months (20), the proportion of women and men reporting comprehensive knowledge of HIV/AIDS prevention (21) and the proportion of men and women using condoms with extra-marital partners (21).  Given our current estimates of population size, for a study involving 16 villages per arm (since power calculations were done separately for men and women) and 40 households per village with a total of 2544 women and 2544 men, and an intracluster correlation of 0.2, the minimum detectable effect sizes for key indicators with 90% power at a 10% significance level are presented below. To allow for 25% of the sample to decline or be lost to follow up, the study will initially sample 53 households per village for a total of 3392 women and 3392 men in the baseline survey. The minimum detectable effect sizes range from 15% to 45%, but are generally between 25% and 30%.   1. **Data Collection Tools and Data Analysis Plan**   Outcome data will be collected by trained enumerators in all villages (both treatment and control) at baseline and 18 months post-baseline. The **“Baseline questionnaire for female respondents”** will be designed and piloted by adapting the “**Pilot questionnaire for Female Respondents**” used in our previous pilot study as well as the WHO Multi-country study questionnaire (1) for questions related to violence and the Ethiopia Behavioral Surveillance Survey of 2005 for other health questions including on HIV/AIDS (21). It will include questions on attitudes and beliefs about gender issues, as well as emotional, physical and sexual violence by an intimate partner in the previous year and during her lifetime. It will also include questions to assess knowledge, attitudes and behaviors related to HIV/AIDS, questions about general health and wellbeing, and a screen for depression using the Patient Health Questionnaire (PHQ), which is currently being validated for the Ethiopian context.  The “**Baseline questionnaire for male respondents”** will be adapted from our “Pilot questionnaire for Female Respondents”, the Ethiopia Behavioral Surveillance Survey 2005 and will include questions on male perpetration of violence as well as HIV/AIDS knowledge attitudes and behaviors, general health and well-being and mental health status. The questionnaires will be translated to Amharic, back-translated to verify the translation and then piloted extensively to ensure that the questions are correctly understood and are appropriate for the cultural context.  In addition to the baseline and endline, there will be ongoing data collection during the implementation of the intervention that provides detailed, real-time feedback on each stage of the curriculum. At each intervention session, 20% of participants will be selected to participate in a brief (eight-question) survey **“Intervention Participants Feedback Survey”** immediately following the session. Participants will be randomly ordered and interviewed in turn each week, such that each is surveyed twice over the course of the 14-week intervention.  In addition, up to 50% of participants will be administered a final feedback survey at the conclusion of the 14-week intervention; this survey is denoted the **Final 7-week questionnaire**. Both questionnaires are identical for male and female respondents. The objective of these surveys is to learn more about participants’ comprehension of the information they will encounter during the intervention. The questions in this survey are a selected number of questions taken directly from the Intervention Participants Feedback Survey.  Finally, enumerators will conduct weekly 30-minute focus groups with selected intervention groups designed to obtain their feedback about the content, structure and usefulness of the curriculum. Focus groups will be conducted using the **“Focus Group Interview Guide”** and will be staggered in timing such that each intervention group participates in only one focus group for the duration of the 14-week course, and 7% of the intervention groups are participating in a focus group each week. All participants in a given group will be invited to attend the focus discussion, which will occur immediately after the intervention. The exact format of the questions to be raised by the enumerator in each focus group will be determined during piloting, but the discussions will focus on reactions to the intervention and the session just conducted; questions about what information or discussion was useful and what was not.  The endline surveys are closely modeled on the baseline surveys. The only notable differences are the omission of sections including questions about socioeconomic status, as these questions do not need to be repeated; and the addition of some questions about exposure to the intervention. The “Endline survey for men” and “Endline survey for women” will be administered to the original baseline respondents. The “Endline survey for men – shorter” and “Endline survey for women – shorter” will be administered to the spouses of the baseline respondents.  In addition, a **qualitative data collection module** will be conducted in conjunction with the endline survey. The qualitative data collection entails a more open-ended, unstructured interview conducted by a data collector trained in qualitative data methods with the objective of learning more about individuals’ perceptions of the intervention, their experiences around relationships and intimate partner violence, and the channels through which the intervention may have shaped these experiences. If the respondent provides consent, the interview will be audio-recorded (no video recording will be utilized). Subsequently, the interview will be transcribed (based on the recording, if available, and enumerators’ notes) and analyzed. The qualitative data collection will include a **maximum of 40 in-depth interviews (20 men and 20 women)** who were included in the intervention, and thus have already been surveyed in the quantitative data collection.  13 females and 12 males who have completed 12th grade, have data collection experience and can speak the local language, will be recruited as enumerators for the baseline and endline; 50% of enumerators will be employed during intervention roll-out to conduct continuous surveys and focus groups with intervention participants. The enumerators will complete a 2 week training on data collection, interview techniques, and questionnaire administration, modeled on the enumerator training conducted as part of the WHO Multi-country Study on VAW. The best-performing enumerators will additionally be engaged in the qualitative data collection, and will receive an additional 3-day training on qualitative data methods supervised by the principal investigators.  In addition, we will also conduct limited photography and videography of the intervention and data collection for training purpose only for those individuals who consent. Photography and videography of limited intervention sessions and survey administration will be conducted to serve as inputs to training modules on intervention development and evaluation and on conducting field research. The videography will be overseen by the project PIs and does not pose any additional risk to study participants or other household members, health clinic workers, or store owners, nor does it provide any additional benefit.  The photography and video informed consent form is included with this application for amendment.   - 1. **Outcomes measured**   1) **Violence against women**: % of women experiencing physical and/or sexual abuse in the past year by an intimate partner and the frequency and severity, % of men reporting perpetration of physical, sexual and/or emotional violence against their female partners in the past year and the frequency, % of women experiencing violence during pregnancy.  2) **HIV**: knowledge and attitudes and behaviors related to HIV such as % with comprehensive knowledge on HIV prevention, % reporting multiple partners, frequency of condom use with extra-marital partners.  3) **Gender perceptions, social norms and perceived social norms**: knowledge and attitudes related to gender equity and IPV, perceptions of others’ beliefs and behaviors with respect to IPV and gender.  4) **General wellbeing and psychological status**: reported stress levels, % with depression as measured using the PHQ-9 (Patient Health Questionnaire).  5) **Other health outcomes and behaviors**: % with history of physical injuries, % with chronic health problems, % with reproductive health problems, % with poor pregnancy outcomes, health seeking behavior, health risk behavior.  6) **Cost effectiveness**: the cost of implementing each intervention per household participating will be tracked and compared to the impact on outcomes of interest.  **2) Developing novel measurement approaches for violence against women**  Data collection related to IPV is challenging and most studies rely on self-reported data which, for a subject as sensitive as violence, is subject to significant under-reporting. Also, there are concerns of social desirability bias in self-reports of violence following an intervention. Despite these concerns, the existing literature has largely relied on self-reports of violence (15, 16). This study will pilot some alternative approaches to collect data on IPV including asking the woman separately about both episodes of violence and injuries or accidents sustained over a given reporting period and asking the woman to report about episodes of violence among sisters, co-wives or neighbors. We will also ask women specifically if they have disclosed their experiences of violence before and if so, when and to whom. This will allow us to measure whether there were changes in disclosure rate and account for any differences. Finally, an anonymous picture technique, which has already been used to improve reporting of childhood sexual abuse in Ethiopia (14) will be piloted for assessment of adult physical and sexual violence. |
| **3. Drugs and Devices.** *If the study involves the administration of an investigational drug that is not approved by the Food and Drug Administration (FDA) for the use outlined in the protocol, then the principal investigator (or sponsor) must obtain an Investigational New Drug (IND) number from the FDA. If the study involves the use of an approved drug in an unapproved way the investigator (or sponsor) must submit an application for an IND number. Please attach a copy of the IND approval (new drug), or application (new use.).*  *If the study involves the use of an investigational medical device and COUHES determines the device poses significant risk to human subjects , the investigator (or sponsor) must obtain an Investigational Device and Equipment (IDE) number from the FDA.* |
| **Will drugs or biological agents requiring an IND be used? YES** **NO**  *If yes, please provide details:*  **Will an investigational medical device be used?** **YES** **NO** *If yes, please provide details:* |
| **4. Radiation** *If the study uses radiation or radioactive materials it may also have to be approved by the Committee on Radiation Exposure to Human Subjects (COREHS). COUHES will determine if you need COREHS approval.* |
| **Will radiation or radioactive materials be used? YES** **NO**  *If yes, please provide details:* |
| **5. Diets** |
| **Will special diets be used? YES** **NO**  *If yes, please provide details:* |

### HUMAN SUBJECTS

| **1. Subjects (*that will be consented for this study*)** | |
| --- | --- |
| 1. **Maximum number:** 8000 women   8000 men | **B. Age(s): 18-49 years** |
| C. Inclusion/exclusion criteriaWhat are the criteria for inclusion or exclusion?Women and men fulfilling the following criteria, and their partners if applicable, will be selected to participate in the study: married or cohabitating individuals aged 18-49 years of age and resident in rural kebeles in the study districts for at least the last six months. **ii. Are any inclusion or exclusion criteria based on age, gender, or race/ethnic origin?** *If so, please explain and justify*  The study only includes adults of reproductive age. | |
| **D. Please explain the inclusion of any vulnerable population (e.g. children, cognitively impaired persons, non-English speakers, MIT students), and why that population is being studied.**  No vulnerable population is included. | |
| **2. Subject recruitment** *Identification and recruitment of subjects must be ethically and legally acceptable and free of coercion. Describe below what methods will be used to identify and recruit subjects* | |
| The study will be undertaken in Meska, Mareko, Sedo, and Silte districts, located in the Gurague Zone in the Southern Nations and Nationalities and Peoples Regional State in Ethiopia. Women and men fulfilling the following inclusion criteria, and their partners if applicable, will be selected to participate in the evaluation: married or cohabitating individuals aged 18 to 49 years, and resident in rural kebeles in the study districts for at least the last six months.  Using the list of villages within the peasant associations of the four districts as a sampling frame, 64 villages will be selected using random sampling. The procedure will take into account the distance between villages to minimize contamination between study arms. Households in the 64 villages will be mapped during a partial census and GPS information will be recorded. For participation in the intervention, baseline and post-intervention surveys, households will be selected using simple random sampling at the village level. In total, 53 households in each village will be sampled.  In the women only, couple and control arms, one woman will be selected by lottery method from all eligible women within the household. This woman (and her husband, if applicable) will participate in the intervention or control information session, and either the wife or her husband will be interviewed at baseline and 18 months post-baseline. In the men only study arm, the male household head will participate in the intervention, and either he or his wife will participate in the survey.  For data collection, villages within each study arm will be randomized to a female survey group (16 villages) or a male survey group (16 villages). In the female survey groups, the woman selected for the intervention or the wife of the man selected for the intervention will be interviewed during baseline and endline, whereas in the male survey group, the man selected to participate in the intervention or the husband of the woman selected will be interviewed (see Figure 1). This design complies with the WHO’s ethical guidelines recommending that women and men in the same villages not be interviewed about violence in order to minimize the risk to women of participating (17, 18). At the same time, it allows the evaluation to separately identify the impact of each intervention on both men and women.  While some studies in other countries have interviewed men and women in the same households about violence, this has never been done in the Ethiopian context. We will assess during the pilot study if it is feasible, acceptable and safe to interview women and men in the same households and if it is, then will modify the study design accordingly and submit an amendment to our application for human subjects approval. If it is found to place women at risk or to alter disclosure patterns of violence, the study design will remain unchanged.  Subjects who have been sampled to participate in the study will be visited at their homes. The study purpose and procedures will be explained carefully to each participant. During the initial consent process, the sensitivity of the research topic will be raised to ensure the participant has the opportunity to reflect on any potential risks in participating in research addressing IPV. Consent will be sought under conditions which ensure that the prospective subject has sufficient opportunity to consider whether or not to participate. In addition, during the subsequent administration of the survey, enumerators will be trained to introduce any section with questions about violence carefully, giving the respondent the opportunity to stop the interview or decline to answer these questions.  Participation in any photography and videography will be voluntary. Conditional on informed consent, selection will be based on which study participants are being surveyed on the days the videography team visits the project. | |
| Please attach a copy of any advertisements/ notices and letters to potential subjects | |
| **3. Subject compensation** *Payment must be reasonable in relation to the time and trouble associated with participating in the study. It cannot constitute an undue inducement to participate* | |
| **Describe all plans to pay subjects in cash or other form of payment (i.e. gift certificate)**  No payment for participation in the research will be provided. However, in order to minimize attrition during the intervention, a small in-kind incentive for attendance such as soap valued at $0.50 will be provided to participants attending all the coffee ceremony session at the 7-week mark and as well at the end of the intervention. Certificates will also be awarded to participants completing all 14 sessions.  **Will subjects be reimbursed for travel and expenses?**  No reimbursement will be provided for travel expenses since the intervention and surveying will take place in their villages. | |
| **4. Potential risks.** *A risk* *is a potential harm that a reasonable person would consider important in deciding whether to participate in research. Risks can be categorized as physical, psychological, sociological, economic and legal, and include pain, stress, invasion of privacy, embarrassment or exposure of sensitive or confidential data. All potential risks and discomforts must be minimized to the greatest extent possible by using e.g. appropriate monitoring, safety devices and withdrawal of a subject if there is evidence of a specific adverse event.* | |
| **What are the risks / discomforts associated with each intervention or procedure in the study?**  The primary ethical concern related to this study is the potential for inflicting harm to respondents through their participation in this study. A respondent may suffer physical or emotional harm if a partner finds out she has been talking to others about her relationship with him.  Interviews on sensitive topics such as experiences of violence can also trigger powerful emotional responses in some participants, causing them to relive painful and frightening events. Thus, psychological distress is a risk for some respondents.  There are also potential risks for field staff participating in the intervention and the data collection. These risks include threats to physical safety from unplanned encounters with abusive individuals who object to the study. Furthermore, fieldworkers may face psychological distress from repeatedly hearing women’s stories of abuse.  **What procedures will be in place to prevent / minimize potential risks or discomfort?**   1. Risks of participating in the intervention   Participation in the intervention may entail the discussion of sensitive and potentially controversial questions related to violence, sexual behavior and relationships between men and women; this may be a particular concern in the study arm involving both men and women. To minimize the risks of participating in the intervention, the following steps will be taken.   1. First, a community advisory board will be established to consult on the development and implementation of the intervention and evaluation. This board will include representatives of the MOH, HAPCO, local women’s organizations, religious organizations, health providers, social organizations and other relevant stakeholders; the investigator at the Addis Ababa University will serve as chair. The board will provide feedback on the proposed curriculum and research and suggest strategies to reduce risk. Following the project initiation, a board meeting will be convened at least every six months to update on progress. Collaboration with a local community advisory board has been a valuable strategy in other GBV studies in South Africa (19). 2. Participation in the intervention will be voluntary, and no one will be pressed during the sessions to discuss personal details that s/he is unwilling to reveal. 3. Study facilitators will be trained to provide referrals to a psychiatric nurse at a local health facility for any participant who requests such a referral. 4. The facilitators will be well trained in minimizing risk to participants and in ensuring their safety and in dealing with potentially dangerous situations. 5. Safety protocols will be in place to protect the participants and the facilitator from harm. These might include having a vehicle and other project staff at the project headquarters on call in case protection is needed. 6. Risks of participating in the survey   In conducting research on violence against women, the safety, privacy and confidentiality of the survey participants must be paramount. In accordance with WHO guidelines on conducting research on VAW (17, 18) the following steps will be taken to minimize the probability that any woman surveyed is put at greater risk of violence by virtue of participation in the study.   1. Interviews will be conducted only in a private setting and will not be conducted in the presence of any other individual over the age of 2 years. Enumerators will be extensively trained on the importance of a confidential setting and tested to ensure that they recognize what settings can and cannot be considered confidential; they will also be trained to reschedule interviews whenever a confidential setting cannot be obtained. Brief refresher trainings on the importance of confidentiality will be conducted monthly for the duration of the study. 2. Enumerators will be trained to terminate or change the subject of discussion if a survey is interrupted, and will be provided with a short diversionary questionnaire they can turn to if the interview is interrupted. Following the diversionary questionnaire, they will terminate the interview and attempt to reschedule to conclude the administration of the confidential portion of the survey. 3. In any public discussion or description of the survey, it will be framed as a study of health and life experiences in this region. 4. Referrals   As per the WHO guidelines for conducting research on VAW, women who report abuse will receive information and referrals for care and support (17, 18). A psychiatric nurse will be employed at the local primary health center for the duration of the project to provide counseling and referral to other services for victims of VAW free of cost.   1. Confidentiality   Strict confidentiality will be ensured throughout the study. In line with WHO guidelines, no names will be written on questionnaires. To enable follow-up data collection, names will be separately recorded on an identification card with a unique code to designate the survey. The identifying information and hard copies of the surveys will be locked in a cabinet and destroyed 5 years after study completion. The electronic database will be password protected.   1. Risks to enumerators and research team members   Past experience on research in VAW has highlighted that all research team members require specialized support and training beyond that normally offered to field enumerators. In light of this, enumerators will participate in monthly day-long refresher trainings and debriefings led by the field research managers and with the participation of the psychiatric nurse engaged by the study. This will provide an opportunity for enumerators to discuss what they have heard in surveys, their feelings about their work and how it is affecting them. It will also enable the research management team to continually reinforce key protocols regarding confidentiality and the protection of respondents’ safety. Enumerators will also be given the opportunity to discuss any concerns with the nurse in private, free of cost, and will have the opportunity to withdraw from the study without penalty.  The videography and photography will be conducted in private. It will be made clear to respondents that the photography and videography is entirely voluntary and they may decline without fear of negative consequences. Informed consent will be sought separately for the videography and photography. | |
| **5. Potential benefits** | |
| **What potential benefits may subjects receive from participating in the study?**  Women and their spouses will benefit from the information about reproductive health, HIV, healthy relationships and violence against women and from the conflict resolution skills acquired during the intervention sessions. What potential benefits can society expect from the study? While a number of interventions to address intimate partner violence have been initiated, few approaches aimed at preventing or responding to IPV have been rigorously evaluated. To date, there have only been two community-based randomized controlled trials of interventions to reduce violence against women and promote gender equity. The study will add to the limited evidence available on interventions to address violence against women and HIV and promote the widespread adaption of effective public health programs that can address these pressing problems. | |
| **6. Data collection, storage, and confidentiality** | |
| **How will data be collected?**  Data will be collected during visits by trained enumerators at subjects’ homes using paper questionnaires at baseline and endline (18 months post intervention). The enumerators will sign a confidentiality form after being trained in confidentiality of data, data security, proper treatment of subjects and preparedness for adverse events. The enumerators will also receive extensive training in line with the WHO guidelines on conducting research on VAW on specific issues regarding confidentiality, the use of diversionary questionnaires in the event of survey interruption.  Data will also be collected during the implementation of the intervention. At the end of each intervention session, 20% of participants will be randomly selected to participate in a very short (5 question) paper-based survey. These surveys will be conducted in a private location to ensure confidentiality. In addition, enumerators will conduct weekly 30 minute focus groups which will occur following an intervention session.  Videography and photography may at times take place outside of the home with maximum effort to de-identify specific location.  **Is there audio or videotaping? YES**  **NO** ***Explain the procedures you plan to follow.***  There will be digital audiotaping of the focus group sessions which will later be transcribed. Any identifying information such as names will be removed from the transcript. Copies of the recording will be kept in a secure, password protected computer and will be accessible only by the PIs of the study. The informed consent for the focus groups will specifically state that recordings will be taken and who will have access to the recordings.  In addition, a videography team led by the project PIs will visit the project site at various timepoints to photography and film intervention implementation and data collection for inputs into e-learning modules around intervention development and evaluation and as well for dissemination purposes. The exercise may include photography and videography of the surrounding area.  Qualitative Interviews (2017):  If the respondent provides consent, the interview will be **audio-recorded** (no video recording will be utilized). Subsequently, the interview will be transcribed (based on the recording, if available, and enumerators’ notes) and analyzed.  **Will data be associated with personal identifiers or will it be coded?**  **Personal identifiers**  **Coded**  ***Explain the procedures you plan to follow.***  The data will not contain any personal identifiers. Each respondent will be assigned a numeric study identifier, and this will be recorded on the survey.  **Where will the data be stored and how will it be secured?**  Hard copies of the survey data will be stored in a locked cabinet at the Addis Ababa University. The soft copies will be kept in password protected computers. Data will be backed up daily on a secure server.  **What will happen to the data when the study is completed?**  The hard copies of the survey will be destroyed by a shredder 3 years after completion of the study. Soft copies will be kept in password protected computers. Identifiers will be encrypted and kept secure by the study PIs.  **Can data acquired in the study affect a subject’s relationship with other individuals (e.g. employee-supervisor, patient –physician, student-teacher, family relationships)?**  Some of the data collected from subjects could conceivably affect their relationship with their partners or their parents. To avoid this, field workers will ensure complete privacy during the interview and will keep all information obtained in the strictest of confidence. Hard copies of the data will be kept in a locked room. | |
| **7. Deception** *Investigators must not exclude information from a subject that a reasonable person would want to know in deciding whether to participate in a study.* | |
| Will information about the research purpose and design be withheld from subjects? **YES**  **NO** ***If so, explain and justify.*** | |
| **8. Adverse effects.** *Serious or unexpected adverse reactions or injuries must be reported to COUHES within 48 hours. Other adverse events should be reported within 10 working days.* | |
| What follow-up efforts will be made to detect any harm to subjects and how will COUHES be kept informed?  The field workers will spend as much time as necessary in each respondent’s home to listen to concerns they may have. Survey staff will be instructed to immediately inform researchers of any adverse effects. In the unlikely event of adverse effects, COUHES will be notified by the researchers within 48 hours. Subjects will also be given contact numbers to study personnel and to COUHES. | |
| **9. Informed consent.** *Documented informed consent must be obtained from all participants in studies that involve human subjects. You must use the templates available on the COUHES web-site to prepare these forms. Draft informed consent forms must be returned with this application. Under certain circumstances COUHES may waive the requirement for informed consent.* | |
| Attach informed consent forms with this application. | |
| 10. The HIPAA Privacy Rule. *If your study involves disclosing identifiable health information about a subject outside of M.I.T., then you must conform to the HIPAA Privacy Rule and complete the questions below. Please refer to the HIPAA section, and to the definitions of protected health information, de-identified data and limited data set on the COUHES web-site.* | |
| **Do you plan to use or disclose identifiable health information outside M.I.T.?**  **YES**  **NO**  *If YES, then the subject must complete an Authorization for Release of Protected Health Information Form. Please attach a copy of this draft form. You must use the template available on the COUHES web-site.*  *Alternatively, COUHES may grant a Waiver of Authorization if the disclosure meets criteria outlined on the COUHES web-site.*  **Are you requesting a Waiver of Authorization?**  **YES**  **NO**  *If YES, explain and justify.*    **Will the health information you plan to use or disclose be de-identified?**  **YES**  **NO**  **Will you be using or disclosing a limited data set?**  **YES**  **NO**  *If YES, then COUHES will send you a formal data use agreement that you must complete in order for your application to be approved* | |

1. Investigator’s Assurance

| **I certify the information provided in this application is complete and correct**  **I understand that I have ultimate responsibility for the conduct of the study, the ethical performance of the project, the protection of the rights and welfare of human subjects, and strict adherence to any stipulations imposed by COUHES**  **I agree to comply with all MIT policies, as well all federal, state and local laws on the protection of human subjects in research, including:**   - **ensuring all study personnel satisfactorily complete human subjects training** - **performing the study according to the approved protocol** - **implementing no changes in the approved study without COUHES approval** - **obtaining informed consent from subjects using only the currently approved consent form** - **protecting identifiable health information in accord with the HIPAA Privacy Rule** - **promptly reporting significant or untoward adverse effects** |
| --- |

Signature of Principal Investigator _______________________ Date __________

**Print Full Name and Title __Vandana Sharma, Research Scientist______________**

**Signature of Department Head __________________________ Date __________**

**Print Full Name and Title ___________________________________________**

***The electronic file should be sent as an attachment to an e-mail:*** [***jadams@mit.edu***](mailto:jadams@mit.edu) ***. In addition, two hard copies (one with original signatures) should be sent to the COUHES office: Building E25-Room 143B.***

1. MIT key personnel all individuals who contribute in a substantive way to the execution and monitoring of the study at or on behalf of MIT or affiliated institutions. Typically, these individuals have doctoral or other professional degrees, although other individuals may be included. In particular, investigators and staff involved in obtaining informed consent are considered key personnel. [↑](#footnote-ref-2)
